# Between the Atolls: Sea Turtle Nesting in the Maldives from 2018 to 2024

**DOI:** 10.3390/ani16020307

**Published:** 2026-01-19

**Authors:** Isha Afeef, Jane R. Lloyd, Ibrahim Inan, Emily Mundy, Martin Stelfox, Stephanie Köhnk

**Affiliations:** 1 Olive Ridley Project-Maldives, H. Kaneerumaage, Dhon Huraa Goalhi, K. Male’, Maldives; gaadhoo@oliveridleyproject.org; 2 Olive Ridley Project, 91 Padiham Road, Sabden, Clitheroe BB7 9EX, UK; jane.lloyd@oliveridleyproject.org (J.R.L.); emily.mundy@oliveridleyproject.org (E.M.); martin@oliveridleyproject.org (M.S.); stephanie@oliveridleyproject.org (S.K.)

**Keywords:** hawksbill, green turtle, olive ridley, Indian ocean, hatching success, incubation time, remigration interval

## Abstract

Sea turtles are a vital part of the marine ecosystem in the Maldives. While five species have been recorded in-water in the country, only two have been historically known to nest here: hawksbills and greens. In this study, we present cumulative data from targeted nest monitoring in combination with ad hoc reports from citizen scientists and marine biologists from 2018 to 2024, with the aim of providing the first comprehensive multi-year dataset on sea turtle nesting activity in the Maldives. Sea turtle nests were recorded from nearly all atolls. The majority were laid by green turtles (n = 1086), in addition to a small number of hawksbill (n = 47), olive ridley (n = 14) and unknown species (n = 65) nests. Nesting occurs year-round, with a peak during the southwest monsoon season from June to September. Median hatching success rate was 90.91% (SD = 15.87) and incubation lasted a median of 59 days (SD = 4.79). Nest inundation and illegal take were identified as the main threats to sea turtle nests, while incidences of egg predation were an exception. The findings of this study will help to clarify conservation priorities and management strategies for sea turtles in the Maldives.

## Simple Summary

Sea turtles are an important part of the marine environment in the Maldives, and they are known to reproduce in the country. With the help of citizen scientists and a dedicated team, sea turtle nesting was documented from 2018 to 2024. The main species nesting in the country was the green sea turtle, with additional nests observed from hawksbills and olive ridleys. Sea turtles nested year-round, with a peak from June to September. Nests laid on beaches throughout the Maldives took around 59 days to hatch, with over 90% of the eggs hatching successfully in most nests. Flooding during high tides and extreme weather conditions together with illegal take were the main threat to sea turtle nests.

## 1. Introduction

The Republic of Maldives is a low-lying archipelago in the equatorial Indian Ocean, with over 1200 small islands across 26 natural atolls [1] famous for their marine biodiversity. The larger atolls have water depths of 30–80 m on the inside, averaging 40–60 m [2], increasing in depth from north to south towards the equator. The northern atolls have a patchier distribution of islands than the south, with more continuous atoll rims [3]. The archipelago’s north and central atolls (from Haa to Laamu) form two parallel, relatively continuous north–south chains with an internal basin with water depths between the atoll chains of up to 600 m [3]. The country’s reefs represent the seventh-largest reef system in the world [4] and are part of the atoll chain extending from Reunion Island to Lakshadweep, India.

The Maldives experiences the Asian monsoon season, with a seasonal reversing of the wind system. The northeast monsoon from December to April is relatively dry with minimal rainfall and calm sea conditions. The intermonsoonal period from May to June has variable weather with thunderstorms and rough seas. June–November is the southwest monsoon, when winds shift predominantly to blow from the southwest, bringing in more frequent rainfall, rough seas and less stable weather—despite rainfall, the temperature remains warm. November–December marks the transition to the northeast monsoon [5].

Out of seven species of sea turtles in the world, five are recorded in the Maldivian waters, including green turtles (*Chelonia mydas*), hawksbills (*Eretmochelys imbricata*), olive ridleys (*Lepidochelys olivacea*), loggerheads (*Caretta caretta*) and leatherbacks (*Dermochelys coriacea*) [6,7,8]. The two most commonly observed species are hawksbills and green turtles, which populate the reefs and seagrass meadows of the archipelago [7,8]. Olive ridley turtles are rarely observed along the reefs or while nesting but are most commonly encountered stranded or entangled in ghost gear [8,9,10]. These three species are also the only ones that have been recorded nesting in the country [8].

There are written accounts of sea turtle egg and meat consumption in the Maldives since the mid-20th century [11]. Sea turtles have been partially protected since 1978, and in 2006, the government imposed a 10-year ban on harvesting sea turtle eggs from 14 nesting hotspots [12]. All sea turtle species are fully protected in the Maldives under the Environment Protection and Preservation Act (4/93) since April 2016, which includes the prohibition of capturing, harming or disturbing turtles, trade and pet keeping, research, and rehabilitation without permit, as well as the extraction of turtle eggs or disturbance of nests [12]. Egg and meat consumption still occurs across Maldives [13], as the country’s geography makes it difficult to enforce protections.

Population-monitoring studies based on photo identification of individual sea turtles in the Maldives have recorded 5150 hawksbills and 1725 green sea turtles by the end of 2024 [14]. The majority of these animals (documented in-water, with rare instances of rescued and nesting turtles included) were identified as juvenile or individuals of unknown life stage, with less than 10% of the hawksbill population and 23% of the green turtles classified as adults [15,16,17]. Abundance and population trend models based on opportunistic photo identification capture–mark–recapture data suggest that while the in-water hawksbill turtle populations are showing different trends in different parts of the country, the majority seem stable and/or increasing [17]. Green turtle populations have been found to be increasing in all surveyed sites [17]. Despite that, green turtles are classified as endangered and hawksbills as critically endangered on the Maldives National Red List due to significant declines in reported nesting activity over the recent past decades [15,16].

Even though hawksbill turtles are the most common species encountered in-water, nests of this species are not reported as often. Hawksbills nests have been confirmed on 11 different islands across five atolls in the past [8], with estimated numbers of 2300 nests or higher per year in the 1980s and 1990s [11,18,19]. More recent reports of hawksbill nesting are much more sporadic and did not exceed 20 nests per year for the entire country in the last five reported years [8,15]. Green turtle nests, on the other hand, have been reported from 39 islands in ten atolls [8] and are presumed to be more abundant, with thousands of nests laid every year, as reports from the 1980s and 1990s indicated 515 and 2048 nesting females per year across the Maldives [6,11]. Surveys carried out in 1984 by Frazier et al. [6] requesting island chiefs to estimate past and current nesting numbers already indicated a drastic decline in observed nesting activity at the time, from 10 s to 100 s of nests a night on some islands to then only one or two nests. More recent surveys carried out on eight islands across four atolls estimated a total of 103 nesting green turtles per year [12]. Estimated and observed nest numbers on an identified hotspot nesting site at L. Gaadhoo declined from the 1980s to 2021 [6,16,20].

The overall total nest number for all sea turtle species might be much greater than recorded, since reporting was mostly carried out on an ad hoc basis [8]. To date, the fragmented nature of sea turtle nesting habitats in the Maldives has prevented the implementation and reporting of sustained, large-scale, multi-year nest-monitoring programmes.

This fits within greater overall observations identifying the Indian Ocean as the geographic area with the highest level of data uncertainty for some sea turtle populations in a global comparison [21,22]. For the Maldives, specific data needs were identified in regional and national reports, including, for example, nesting information, genetic stock analysis and investigation of migratory patterns [8,15].

The Olive Ridley Project (ORP) has been collecting ad hoc data on sea turtle nesting activity in the Maldives since 2014, with contributions from citizen scientists and marine biologists [7]. Starting in the end of 2017, ORP has been regularly collecting data from select islands in Baa, Laamu, Lhaviyani, Noonu, North Malé and Raa atolls through the deployment of a permanent team member on-site. This information has been continuously supplemented by citizen science data from various members of the public throughout the years. The aim of this study is to provide a comprehensive multi-year summary of recorded sea turtle nesting activity in the Maldives from 2018 to 2024, comparing nest season variability, nesting success and threats to sea turtle nests across different atolls in the Maldives.

## 2. Material and Methods

### 2.1. Study Area

The Maldives archipelago consists of 1192 sandy islands in a predominantly geographical double chain of 26 natural atolls covering an area greater than 90,000 km^2^ [1], organised in 20 administrative units. Only about 200 of the islands are inhabited, with an additional 174 islands developed and currently operational as resorts for tourism [23].

**Figure 1:**
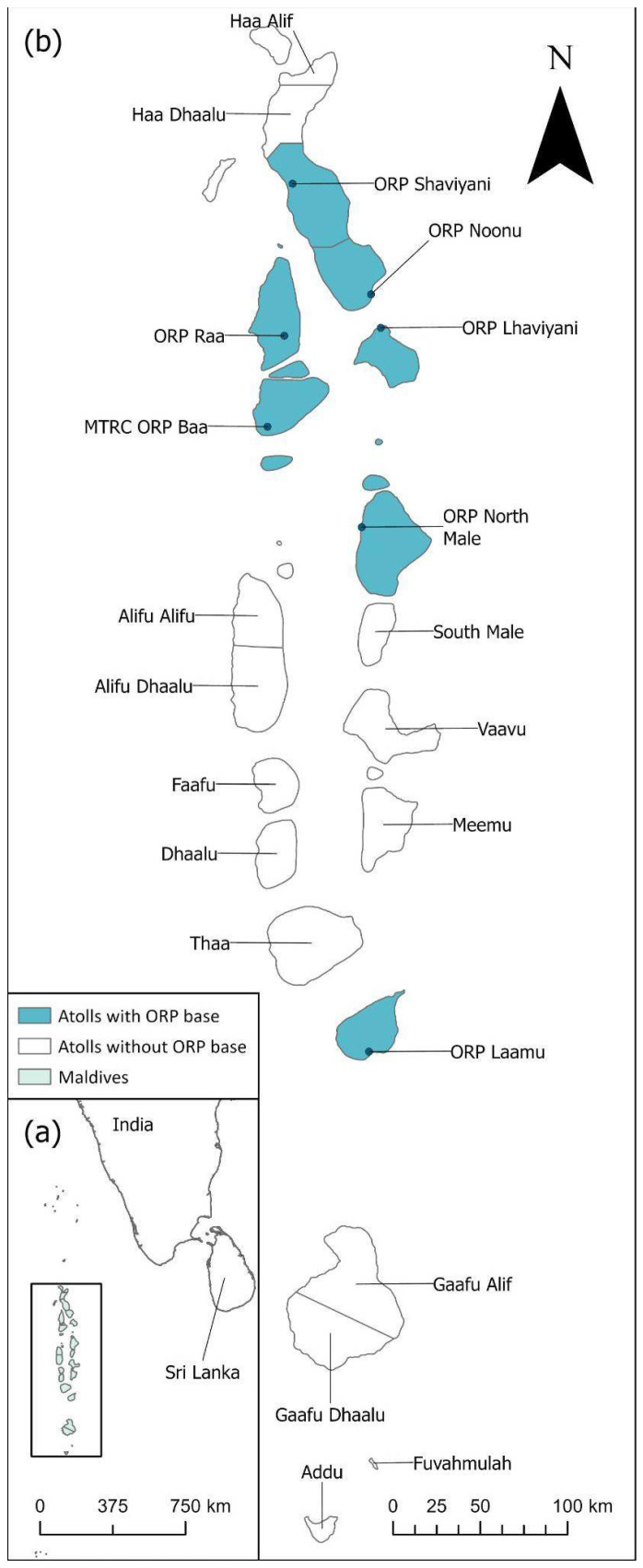
ORP bases in Maldives on a full country map, legend applies to (**a**,**b**). (**a**) The location of the Maldives includes India and Sri Lanka to the northeast. (**b**) ORP base of operations including operational years from north to south, Shaviyani (2022 to date), Noonu (2022 to date), Raa (2021 to date), Baa (2016 to date), Lhaviyani (2017–2024), North Male (2019 to date), Laamu (2018 to date).

Primary data collection points included islands and atolls where ORP team members were present year-round (Figure 1), with the most consistent data collected from resort islands that have marine biology teams, and security performing regular patrols of the island. The majority of data reported is from the central Maldivian atolls of Alifu Alifu and Alifu Dhaalu, North and South Male, and Lhaviyani and Baa, which have the highest density of tourism activity. Ad hoc and regular surveys were also carried out in the uninhabited island of L. Gaadhoo; however, data collection was dependent on access to the island, which is greatly impacted by the weather. Rough weather conditions and higher instances of storms between May and December impact survey activity and pose challenges to data collection.

### 2.2. Data Collection and Analysis

For data reporting, the administrative atoll distinction was followed, except for the Kaafu atoll, where the geographic distinction into the North and South Malé atolls was applied to provide a detailed resolution of the collected information.

General nesting and hatching data for this study was collected by ORP team members and supplemented with information submitted to ORP by citizen scientists following the initial Turtle Watch Maldives initiative [7], from a total of 66 islands across 19 atolls in the country. Interested parties were provided with basic training upon request. Additionally, public online and in-person trainings were conducted on a regular basis as part of the ORP outreach and the Sea Turtle Guardian Programme. Training included the identification of nests, turtle species and tracks, marking and monitoring of nests, hatching and excavation protocols, illegal take identification, and relevant reporting, and followed a sea turtle Nesting Protocol developed by ORP and endorsed by the Environmental Regulatory Authority (former Environmental Protection Agency) Maldives.

Data collection was carried out consistently, except in 2020, when it was affected by travel restrictions related to the COVID-19 pandemic. Initially, tourism and tourism-related activities ceased due to official border closure from 27 March to 15 July 2020 and all inter-island travel in the Maldives was severely restricted to limit the spread of the virus. In the following months, activities resumed slowly across the country, including a slow increase in data submissions.

Trained citizen scientists were encouraged to record false crawls and true nests, their GPS location, nesting species and hatching dates whenever possible. False crawls were recorded when a turtle made no obvious nesting attempt and returned directly to the ocean, or when evidence of digging was apparent, yet successful nesting had not occurred, and no egg chamber could be located. True nests were recorded when egg laying was observed, an egg chamber could be located, and/or hatchlings were observed. Data were mainly obtained from resort islands, due to the presence of marine biologists with expertise in data collection on those islands.

On islands where the ORP team was based, further data was collected when possible, including track width, photographs of females for photo identification (photo ID) purposes [24], measurements of the nesting female, including CCL (curved carapace length) and CCW (curved carapace width). Nest relocations were carried out in case nests that were at risk of inundation or wash out from beach erosion, following established protocols and performed under permits from relevant authorities (Environment Protection Agency: EPA/2022/PS-SP/05, EPA/2023/PS-SP/02, EPA/2023/PS-SP/02). Nests were monitored throughout the incubation period and posthatching nest excavations were conducted by ORP team members 48–72 h after the emergence of the first hatchling, to allow for the natural emergence of any stragglers, following relevant permits (Environment Protection Agency: EPA/2018/PSR-T03, EPA/2020/PSR-T06, EPA/2021/PSR-T14, EPA/2022/PSR-T06, EPA/2023/PSR-T10 and Ministry of Fisheries and Agriculture: NRP2023/35). Nest contents were analysed, including the opening of any unhatched eggs and the classification of egg contents, following previously published protocols adapted from [25,26]. The incubation time for nests was calculated for nests where both the nesting event and the date of first emergence was available, calculating the difference in days between the two. Hatching success was calculated as follows: ((total number of eggs − number of live trapped − number of dead trapped)/total number of eggs) × 100. Emergence success was calculated as follows: ((total number of eggs − number of unhatched eggs)/total number of eggs) × 100.

On L. Gaadhoo, a nesting hotspot in the country [7,12], regular surveys were performed for nest monitoring, following the same procedures as described above, and to identify instances of illegal take. Ad hoc surveys were performed from 2018 to 2022. All data was recorded as “suspected” (i.e., suspected nests and suspected false crawls) when signs of sea turtle digging and subsequent camouflage indicated a nest or nesting attempt, even if neither the presence of eggs, nor evidence of hatching could be confirmed. Nests were recorded as true nests if the tracks were determined to be fresh, i.e., presumably laid within the past two weeks, as estimated by the amount of vegetation or trash covering it and taking into account previous survey dates. Regular surveys and nest excavations commenced in 2023 as part of a newly established Sea Turtle Ranger Programme implemented by the Environmental Protection Agency in cooperation with ORP. Throughout all survey activities, suspected and true nests were reported as illegally harvested when they were associated with evidence of egg removal, such as egg chamber searches with sticks, large man-made conical depressions and/or small clusters of egg fragments.

## 3. Results

### 3.1. Summary

**Figure 2:**
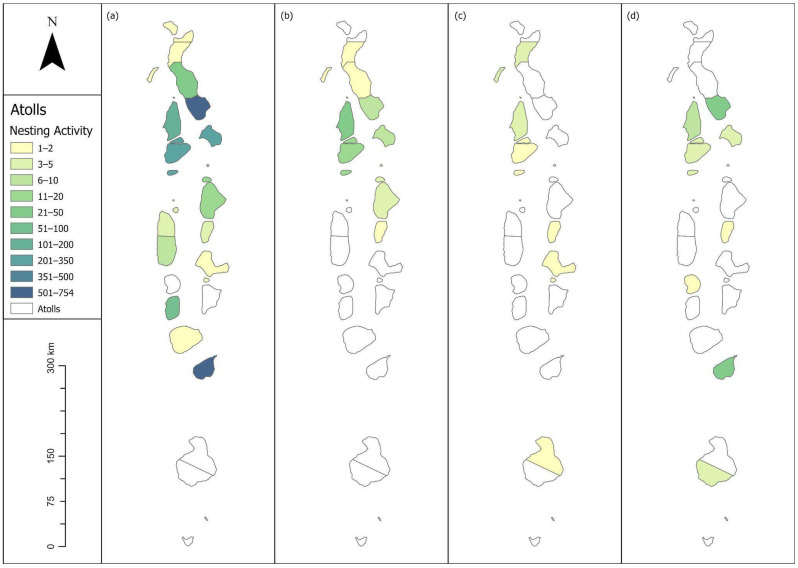
Series of all species nesting encounters throughout the Maldives atolls shaded by the total turtle nesting activity reported. (**a**) Green, (**b**) hawksbill, (**c**) olive ridley, (**d**) unknown species.

A total of 2310 incidents of nesting activity was recorded from January 2018 to December 2024 in the Maldives, out of which 1212 were true nests, 1079 were false crawls, and 19 were unspecified activities. Data was recorded from 18 out of 20 administrative atolls, with data from Kaafu being distinguished into North and South Malé during this period (Table 1, Figure 2).

The majority of nests were laid by green sea turtles across the Maldives, totalling 1086. Only 47 nests were laid by hawksbills, and 14 recorded nests were from olive ridleys. We also recorded 65 nests where the species of the nesting turtle was not reported. Most nests were recorded from Laamu Atoll (n = 434), split between the islands Gaadhoo (n = 288), Olhuveli (n = 144), Fonadhoo (n = 1) and Hulhimendhoo (n = 1); and Noonu Atoll (n = 349), split between the islands of Medhufaru (n = 269), Ehdhuffarumaira (n = 64), Fushivelaru (n = 13), Orimasvaru (n = 2) and Orivaru (n = 1).

Flooding and inundation, illegal take and predation were documented as factors negatively impacting nests during incubation. Out of the 1212 nests, 63 nests were fully or partially inundated or flooded throughout the incubation period (5.2%). A total of 133 nests showed signs of human interference (10.96%), such as unauthorised excavation, holes poked in the sand or fragments of broken eggs in combination with human tracks. Out of these, all but one nest were located on non-resort islands. A further 23 false crawl sites and 11 of unspecified nesting activity showed signs of human interference, bringing the total to 167 (13.8%). A total of 29 nests showed signs of predation from either crabs or ants (2.4%).

A large proportion (69.6%) of true nests were recorded by ORP staff (n = 844), with a total of 103 citizen scientist contributors providing data for 315 true nests. There were also 50 true nests with no observer data available.

### 3.2. Olive Ridley

Solitary nesting of olive ridley turtles has been reported from six atolls in the Maldives; predominantly in northern atolls Haa Dhaalu (n = 5), Baa (n = 1), Raa (n = 4), and central atolls and South Malé (n = 1), Vaavu (n = 1), with the exception of two nests recorded in Gaafu Alifu atoll (Table 1). The 14 true nests were recorded in December to February, with one exception where a nest was recorded in August in the Raa atoll (Figure 2c). No false crawls or failed nesting attempts have been reported for this species.

Clutch size varied between 44 and 148 eggs (median = 132, n = 5, SD = 41.05), with a hatching success rate of 16.8–93.9% (median = 62.8%, n = 5, SD = 32.96) reported after an incubation time of 50–66 days (median = 60, n = 4, SD 7.39). A total of 319 hatchlings were observed from five nests in total (min = 18, max = 118, median = 59, SD = 39.12).

### 3.3. Hawksbill

**Figure 3:**
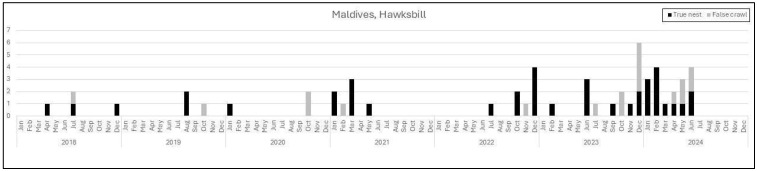
Stacked chart of true nests (black) and false crawls (grey), by year by month for hawksbill nesting activity (all Maldives).

A total of 65 incidents of nesting were recorded in the study period, 47 of which were true nests. The majority of hawksbill nests have been recorded in the Northern Atolls of Maldives, in Raa (n = 14), Baa (n = 13), and Noonu atolls (n = 7). Further nests were reported in the Upper North Province (Haa Dhaalu n = 2, Shaviyani n = 2) and the North Central Province (North Malé n = 3, South Malé n = 1) (Table 1, Figure 2b). False crawls were also only reported from the North of the country (Baa n = 6, Lhaviyani n = 1, Noonu n = 3, North Male n = 1, and Raa n = 7). No specific nesting season has been identified for hawksbill turtles, with nests being reported sporadically, but throughout the year (Figure 3).

Clutch size was recorded for 26 nests ranging from 68 to 180 eggs (median = 128, SD = 26.66). After an incubation period of 52 to 70 days (median = 60.0, n = 17, SD = 5.55), nests hatched with a hatching success rate of 39.44–100.0% (median = 88.05%, n = 26, SD 17.56). A total of 2722 hatchlings were recorded, ranging from 45 to 157 per nest (median = 106, n = 26, SD = 28.51).

### 3.4. Green

#### 3.4.1. Nesting Distribution

**Figure 4:**
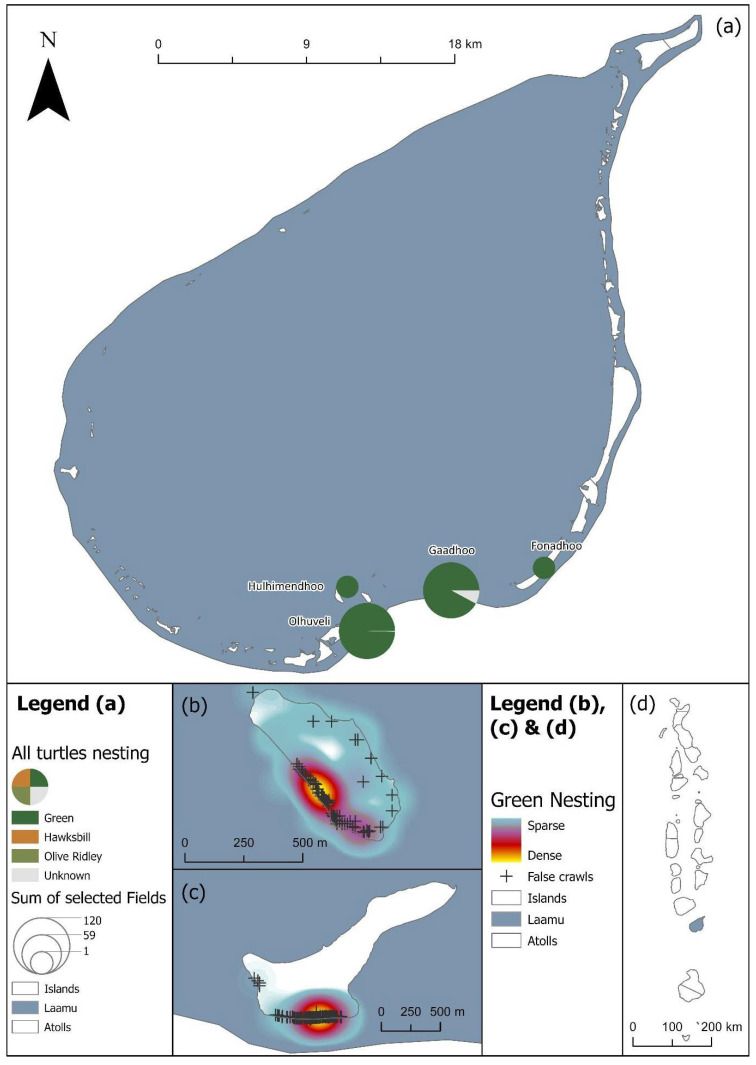
(**a**) Laamu’s turtle nesting activity as scaled pie charts by species for each island. (**b**) Green turtle nests on Olhuveli island are displayed as a heatmap with false crawls marked. (**c**) Green turtle nests on Gaadhoo island are displayed as a heatmap with false crawls marked. (**d**) Location of the Laamu atoll within the Maldives, with nesting activity shaded by number of nesting encounters (as shown in Figure 2a). See legend for scale and symbology.

**Figure 5:**
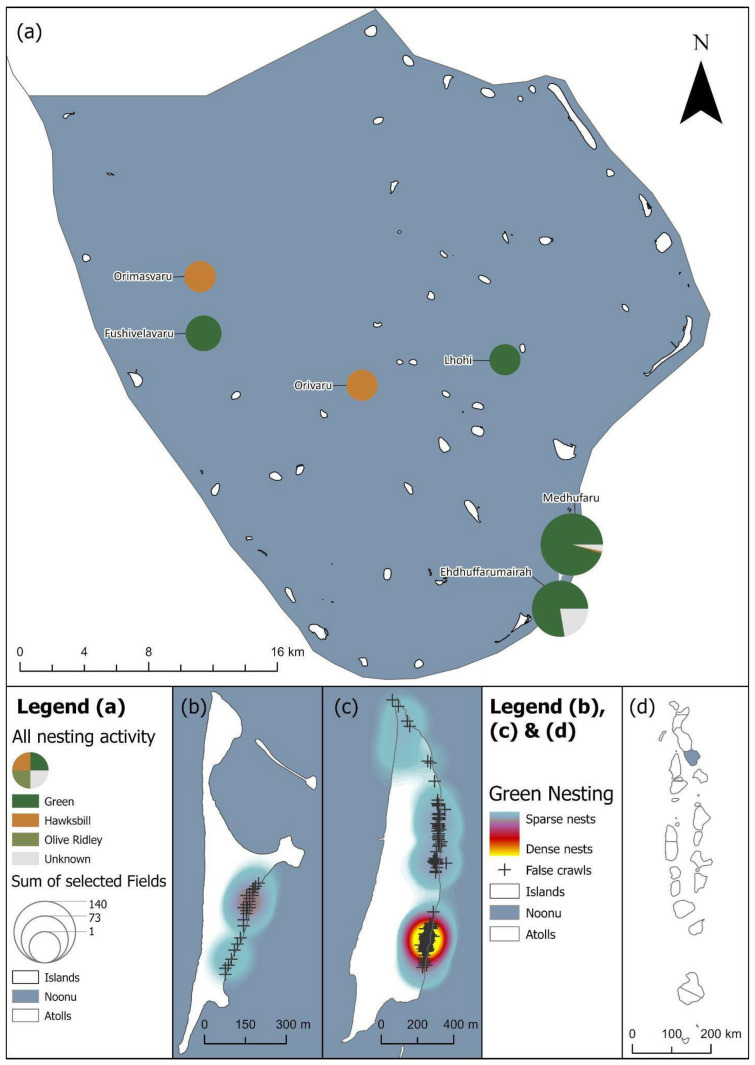
(**a**) Noonu’s turtle nesting activity as scaled pie charts by species for each island. (**b**) Green turtle nests on Ehdhuffarumairah island are displayed as a heatmap with false crawls marked. (**c**) Green turtle nests on Medhufaru island are displayed as a heatmap with false crawls marked. (**d**) Location of the Noonu atoll within the Maldives with nesting activity shaded by number of nesting encounters (as shown in Figure 2a). See legend for scale and symbology.

**Figure 6:**
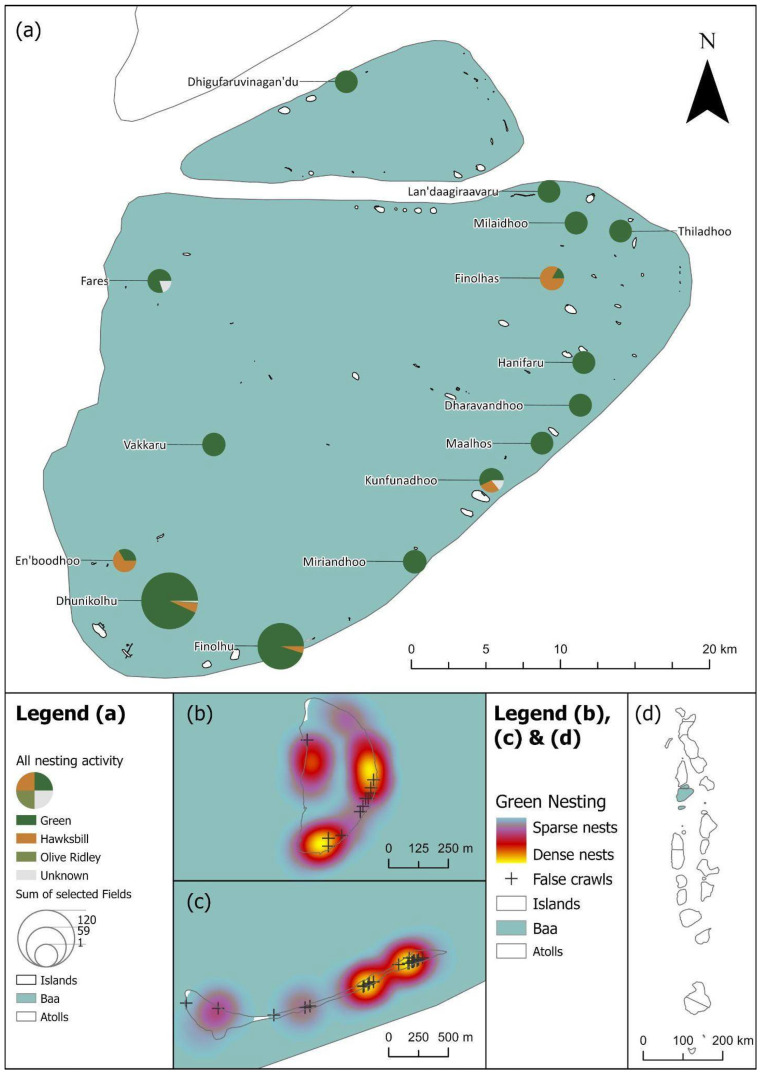
(**a**) Baa’s turtle nesting activity as scaled pie charts by species for each island. (**b**) Green turtle nests on Dhuni Kolhu island are displayed as a heatmap with false crawls marked. (**c**) Green turtle nests on Finolhu island are displayed as a heatmap with false crawls marked. (**d**) The location of Baa atoll within the Maldives with nesting activity shaded by number of nesting encounters (as shown in Figure 2a). See legend for scale and symbology.

**Figure 7:**
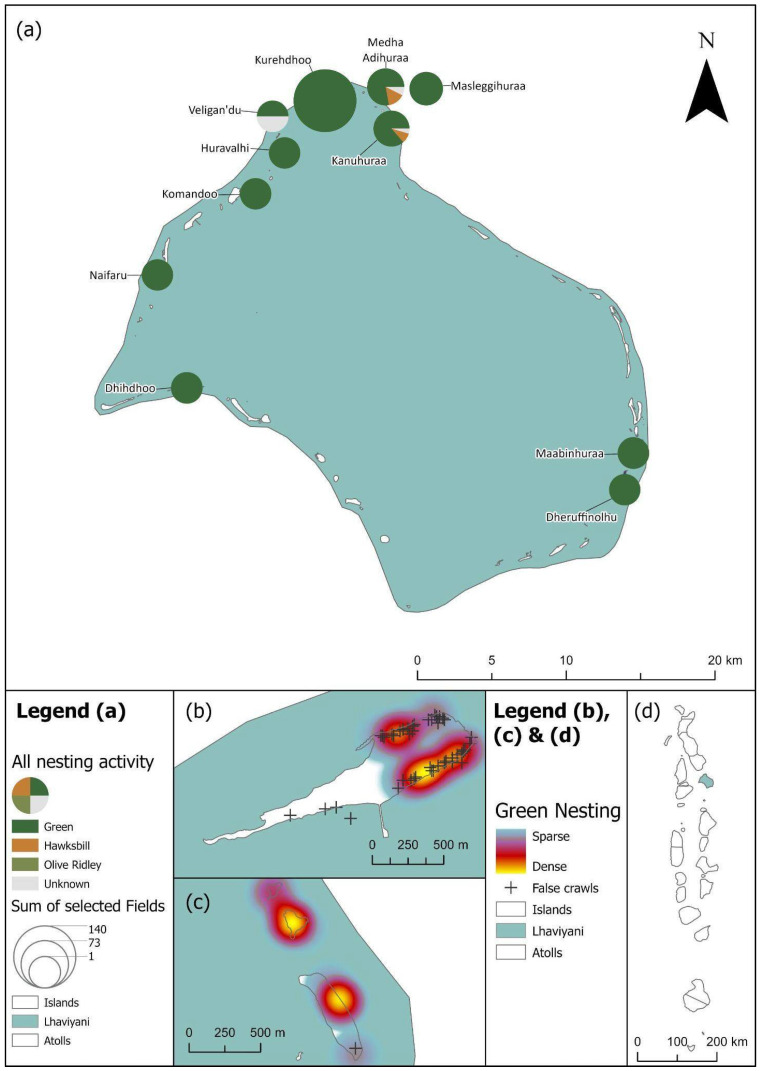
(**a**) Lhaviyani’s turtle nesting activity as scaled pie charts by species for each island. (**b**) Green turtle nests on Kurehdhoo island are displayed as a heatmap with false crawls marked. (**c**) North to south Masleggihuraa and Medha Adihuraa islands with green turtle nests are displayed as a heatmap and false crawls are marked. (**d**) Location of Lhaviyani atoll within the Maldives with nesting activity shaded by number of nesting encounters (as shown in Figure 2a). See legend for scale and symbology.

A total of 1086 true nests, 1030 false crawls and six unknown green sea turtle nesting activities were recorded throughout the study period across 16 atolls in total (Table 1, Figure 2a). The majority of true nests were recorded in Laamu (n = 409, Figure 4), Noonu (n = 316, Figure 5), Baa (n = 117, Figure 6) and Lhaviyani atolls (n = 110, Figure 7).

The most nesting encounters were recorded in Noonu (n = 755). In this location, survey efforts have changed over time. Since 2018, citizen scientists have submitted data on sea turtle nesting activity (n = 22), with no nesting data submissions in 2019 and 2020. Citizen scientist submissions resumed in 2021 (n = 41). ORP nesting survey efforts started in January 2022 with a staff member based on Medhufaru island, resulting in more comprehensive and complete data collection.

#### 3.4.2. Nesting Seasonality

**Figure 8:**
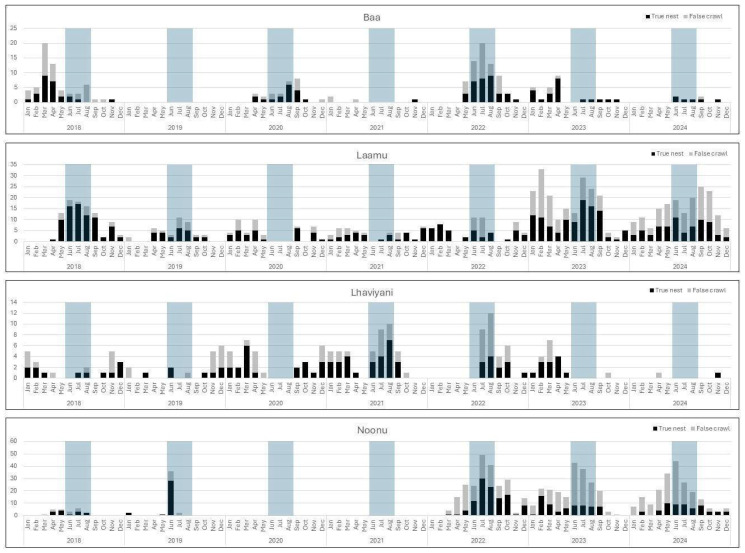
Series of stacked charts of true nest and false crawl activity, by year and by month, for Baa, Laamu, Lhaviyani and Noonu atolls (top to bottom). Blueshaded area indicates peak months for nesting activity averaged across all of the Maldives.

No true nesting period was identified for green turtles across the Maldives between January 2018 and December 2024; however, nesting activity showed a peak during the months of June to August. Overall, the temporal distribution of nesting varied across the atolls and across the years (Figure 8). From the four atolls with highest turtle nesting activity (>150 nesting activities overall), Noonu showed the clearest distinct peak with the highest number of nesting activity typically in June to August. In Laamu atoll, across all years, nesting was recorded relatively consistently throughout the year, with peaks in July to September, and smaller peaks in January to March. The expected mid-year peak was not observed in 2021 despite continued monitoring efforts. For Baa atoll, no clear pattern could be identified, with peaks in nesting activity varying between individual years, ranging from March to April as well as June to August. Nesting activity in Lhaviyani atoll seemed to follow a contradictory trend in contrast to Noonu atoll, with peak nesting often reported from October to March. Again, in 2021, this pattern was reversed and the peak in nesting activity occurred from June to September.

#### 3.4.3. Clutch Size, Incubation Period and Hatching Success

Green turtle nests across the country ranged in clutch size from 2 to 205 eggs (median = 103, n = 459, SD = 28.74), with the majority of nests with a clutch size of larger than 70 eggs (n = 421). The remaining clutches showed unusually low egg counts for green turtles; some were reported to be the result of interrupted nesting (n = 1), the inundation events recorded (n = 8) and/or erosion (n = 1), as well as predation (n = 1) of the nest prior to excavation. For nests for which both nesting and emergence date have been recorded, the median incubation time was 59 days (SD = 5.15, MIN = 46 days, MAX = 76 days, n = 288). Hatching success rates for green turtle nests ranged from 0.0 to 100.0% (median = 90.91%, Table 2), based on the excavation of 459 nests. A total of 19 nests showed a hatching success rate of 100%, 206 of over >90%, and six nests where all eggs failed to develop (Supplementary Table S1). Overall, 39,024 green turtle hatchlings were recorded (n = 494), with a median of 85 hatchlings per nest (SD = 36.09).

#### 3.4.4. Inter-Nesting and Remigration Intervals

**Figure 9:**
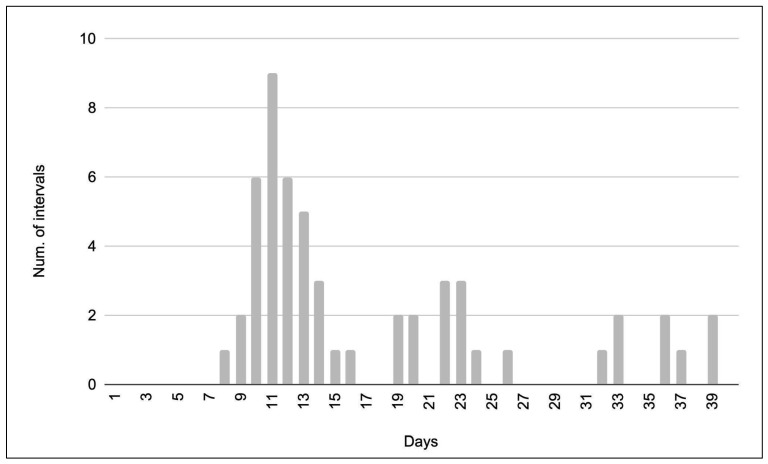
Observed inter-nesting intervals in days for females observed nesting more than once per nesting cycle.

In total, 44 different nesting female green turtles were identified using photo ID throughout the study period. Out of these, 20 individuals were encountered more than once, with five individuals being observed in more than one individual nesting season. Intervals between resightings (i.e., recaptures) within one nesting season ranged from 8 to 39 days (Figure 9), with intervals clustering in three distinct waves around 8–16, 19–26 and 32–39 days. For analysis of median inter-nesting intervals, we only included the first wave of resightings, as the second and third wave most likely resulted from missed clutches (see [27] for reference). Thus, the median inter-nesting interval was found to be 11 days (n = 24).

For the five turtles that were observed in more than one nesting season (i.e., across multiple nesting seasons for the same individual), the median remigration interval was 2 years, 9 months, and 29 days (34.5 months). One turtle was documented in three separate nesting seasons, with remigration intervals ranging from 2 years, 6 months, and 14 days (30.43 months) to 1 year, 8 months, and 2 days (20.07 months).

Interestingly, the nests laid by this individual in her third nesting season showed significantly low hatching success rates of only 9.68 and 6.00%, respectively, which is in contrast to the nests laid in the two previous seasons, where hatching success ranged from 73.08 to 100%. No external factors, such as predation or flooding, were recorded during the third nesting season, indicating an internal source of developmental failure, such as inadequate nutritional status of the female or fertilisation failure.

#### 3.4.5. Threats and Nest Relocations

The primary threats to sea turtles’ nests which prevented them from successful development and hatching were illegal take (n = 133), inundation and/or erosion (n = 63) and predation (n = 28).

Illegal take of nests posed the highest risk factor to sea turtle nests in the country, according to the currently available data. Over the study period, 133 nests showed signs of human interference indicative of illegal take, such as footprints, digging, and holes showing nest search behaviour. The nests were located mainly on L. Gaadhoo (n = 86) and N. Ehdhuffarumairah (n = 34), both of which are islands with some of the highest number of documented nests in the Maldives overall. The majority of nests subject to illegal take were laid on non-residential islands at 93.18% (n = 123), with only a small number of nests reported as illegally taken from resorts at 6.06% (n = 8) or residential islands at 0.76% (n = 1).

In total, 63 nests were impacted by the threat of inundation and/or erosion, with 60 nests being washed over, inundated or eroded away. To mitigate the threat of inundation and/or erosion, nests were relocated following regulations from the EPA Maldives. Nests were relocated due to imminent risk of flooding (within 1 m above or below the hightide line), risk of inundation due to location on a known flood zone, previously recorded or observed beach erosion or unique cases of nest vulnerability (e.g., due to placement on a road) (Supplementary Table S1). Nest relocations were subject to case-by-case permits granted by the EPA Maldives prior to June 2022, resulting in relocations being performed only sporadically (N = 13) until dedicated long-term permits were granted as of June 2022. Following that, until December 2024, another 42 relocations were performed. The total 55 relocated nests represent 4.53% of all nests recorded during the study period. Despite having access to relocation permits, eight nests were nevertheless subject to inundation events due to unforeseen severe weather conditions. The median hatching success for successfully relocated nests was 85.86% (n = 43), which is comparable to the overall national median hatching success.

Out of the 1086 true green turtle nests, 1031 green turtle nests remained in situ for the entire incubation time. A total of 49 of these nests were observed or suspected to have been flooded during the incubation process.

A total of 28 nests showed signs of predation (HK = 1, GR = 27), mainly identifying crabs and ants as the potential predators.

## 4. Discussion

The Maldives archipelago provides a nesting habitat to three different sea turtle species. However, the abundance of sea turtle nesting activity has been greatly understudied due to the highly dispersed nature of this nesting habitat on nearly 1200 islands. As showcased by [7], the combination of targeted, on-site monitoring efforts with the utilisation of citizen science reports provides a great baseline datasource with which sea turtle nesting in this challenging environment can be assessed, and here, we present an initial multi-year dataset highlighting the abundance of and threats to sea turtle nests in the country.

While nesting reports are biassed towards islands with ORP team members on site or resort islands with active marine biology programmes, the citizen science programme also engaged members of the general public, who provided information from residential and non-residential islands. Aiming for an increase in citizen science participation may provide a better coverage of overall detection of nesting activity and enhance the chances of recording rarer nesting species such as hawksbills or olive ridleys. Following the reports from this study, beaches with consistent known nesting activity and reliable monitoring opportunities, such as L. Gaadhoo or B. Dhuni Kolhu, are recommended to be used as index nesting beaches as are commonly used for the assessment of sea turtle nesting trends (see, for example, [28,29]). Especially for L. Gaadhoo, historical nest numbers are available [6,11] and consistent multi-year monitoring has been implemented in recent years [30].

Hawksbill turtles have been known to nest in the country in the past with reported nesting numbers varying greatly in records from the 1980s to 1990s, with a low of 126 and a high of 11,820 reported in 1993 [11]. More recent studies show very low yearly incidences of hawksbill turtle nests [7,8] on up to 11 different islands predominantly located in the northern to central part of the atoll chain. Our findings support these observations, with less than 10 nests recorded every year, mainly in the northern part of the country, which indicates a significant decline in hawksbill turtle nesting since the 1990s, which led to the species being listed as Critically Endangered on the Maldives National Red List in 2022 [15]. High capture rates of turtles as well as nests during the same period [11] might have contributed significantly to the decline of the species.

Olive ridley turtles are known from rare in-water encounters and most frequently from interactions with fisheries and entanglement in ghost fishing gear in the country, with no historical records of nesting of the species [6,9,11]. Since 2018, however, sporadic nesting has occurred in the archipelago [8], and in this paper, a total of 14 true nests from 2018 to 2024 have been reported. All except one of these nests were laid between December and February, partially coinciding with the peak nesting period for the species in Eastern India and Sri Lanka [31,32]. Genetic analysis of olive ridley turtles found entangled in ghost fishing gear and rescued in the Maldives found these turtles to belong to Indian and Sri Lankan stocks [33]. Future genetic studies of olive ridleys nesting in the Maldives may provide insights into the origin of these turtles and a potential nesting range expansion of the species within the Northern Indian Ocean.

Green turtles were found to be by far the most frequently nesting species in the Maldives, which agrees with previously published data [6,8,11]. While the species has recently been uplisted on the IUCN Red List of Threatened Species from Endangered to Least Concern on a global scale due to significant recoveries of certain populations [34], the regional assessments still classify the species as Vulnerable in the Northern Indian Ocean [35] and Endangered in the Maldives [16]. Green turtle nesting has declined from one to two thousand each year in the late 1980s to 1990s [11] to a maximum of 300 nests per year from 2018 to 2024, representing an assumed decrease of 70–78.3% [16]. Similarly to hawksbill turtles, this period also coincides with significant take of turtles as well as nests [11]. Further data collection in the coming years should enable a more up-to-date trend analysis for green turtle nesting numbers, as the current survey period of seven years is too short for the identification of trends due to differences between individual nesting years. Nesting numbers fluctuate between consecutive nesting seasons, in part due to remigration intervals of female turtles, which for green turtles has been found to vary predominantly between two and six years [36,37]. In this study, the majority of nesting females that were sighted in more than one nesting season had a median remigration interval of 2.84 years, thus falling into the common frequency described for this species [38,39].

This study found no clearly defined nesting season in the Maldives, with nesting occurring year-round with a peak in activity towards the middle of the year. Similar nesting behaviour with general year-round nesting activity with distinct peaks in certain months has been reported from turtle populations in Sri Lanka [40], as well as green turtles in northwestern Costa Rica [41].

Most nesting in the Maldives occurs during the wetter southwest monsoon season [5], a phenomenon also reported from some other green turtle rookeries such as on the Caribbean coast of Costa Rica [42,43] or in the Aldabra atoll [44]. Seabrook [44] suspected that beach conditions were preferable for nesting females during the monsoon season, leading to less emergences and abandoned nesting attempts when compared to the dry season. Ref. [45] discusses the potential impact of temperature during high nesting in the rainy season, which coincides with the warmest months of the year. In contrast, low nesting density dry season nests were subject to lower incubation temperatures and might provide important balance for the fitness and sex ratio of hatchlings produced throughout the year [45]. As the Maldives does not experience significant differences in ambient temperature throughout the year, the impact of precipitation on sand temperatures [46] and, therefore, hatchling sex ratios, might be quite substantial and a main driver of variation in incubation conditions.

Although nesting during the rainy seasons seems to come with benefits such as variable sand temperatures, it is not without challenges either. In the Maldives, there is a higher risk of severe weather events such as storm surges during the southwest monsoon, leading to beach erosion or inundation, which this study identifies as the second most common threat to the successful development of the nest. Inundation and prolonged intense precipitation have been associated with decreased hatching success before [47,48], resulting from osmotic stress and/or reduced availability of oxygen to the developing embryo when underwater [49,50,51]. This matches the low hatching success rates observed following inundation events observed in the Maldives. As the lowest-lying country in the world, the Maldives as a whole is at exceptional risk of coastal flooding [52,53] from sea level rises and increased wave heights, which is potentially an increasing risk to sea turtle nests in the country in the future.

Illegal take was the biggest threat to sea turtle nests in the Maldives identified in this study. The use of sea turtle nests has been historically documented [11] and is of past economic and ongoing cultural importance in the country. The harvest of sea turtle eggs only became illegal in the past 20 years [12], posing relatively new restrictions on the harvesting practices in the archipelago. Recent efforts towards a reestablishment and implementation of locally led conservation projects on nesting beaches [30] aims to address this threat in a sustainable way embedded in local culture and management experience. Future management approaches need to include local stewardship to address egg harvest, as enforcement on the dispersed and often uninhabited islands will otherwise continue to be challenging.

The average hatching success rates of green turtle nests in the Maldives was high with a median of 90.5%, which matches globally reported high success rates as found by a recent systematic review [54]. Studies from other nesting populations, for example, in Galápagos, Tanzania and Turkey, reported a similar great variability in hatching success as found in the Maldives [55,56,57]. Overall, the current beach habitat in the country seems well-suited for the successful development of green turtle nests, but this might change as the low-lying island nation continues to face the threat of rising sea levels.

The median incubation time for green turtle nests was found to be 59 days. A recent study on incubation duration trends in Florida showed significantly lower times of just over 50 days for green turtle nests laid in the state in 2022 [58]. As incubation temperature is linked to both the incubation duration and sex ratio of hatchlings, with nest incubation at higher temperatures producing more females and hatching quicker, so that shorter incubation times have been linked to the feminisation of hatchling sex ratios (see, for example [57,59,60]). Thus, the median incubation time of 59 days observed in the Maldives, which is a tropical equatorial nation, may indicate a stronghold for male hatchling production for this species, highlighting its importance despite relatively low nest numbers in comparison to other major nesting beaches around the globe. Future studies into nesting beach characteristics, temperature, female nesting habitat choice, and egg development should be conducted to further investigate the matter and to establish currently unknown factors, such as pivotal temperature, for the Maldivian nesting population.

### 4.1. Limitations

Due to the highly dispersed nature of the archipelago, nest monitoring using regular beach walks or night patrols is extremely resource-intensive and has therefore not been carried out consistently for all islands in the Maldives. The utilisation of drones for distance monitoring is one option to increase geographical coverage but is also still limited due to the range and flight times of commercially available drones.

The current data collection model described in this paper utilises expertise and interested individuals already in place, whether as residents or due to professional engagements, on a variety of islands in the country. This citizen science data collection framework increased the overall geographical coverage for sea turtle nesting information, while specialised personnel increased the temporal resolution on select islands. Due to the ad hoc nature of many nesting activity reports, no correlation between nest numbers and monitoring effort can be established at this point in time. As islands are small, host a limited number of people and often relatively low number of nests per island each year, the implementation of beach monitoring programmes as utilised in other parts of the world has not been possible, logistically and economically, up to this day.

Nesting reports are currently biassed to inhabited islands, including residential and resort islands, with a large part of the country’s uninhabited islands with unknown nesting status. Targeted survey effort during the identified peak nesting period for the country might help identify islands where increased monitoring effort could be of use.

Since the islands in the Maldives are of relatively uniform sandy characteristics, most might provide suitable sea turtle nesting habitats, with relatively low nest numbers for each individual island. A more detailed habitat characterisation of currently identified nesting hotspots, e.g., regarding beach slope, beach vegetation and sediment characteristics, might narrow down habitat suitability for islands of currently unknown nesting status.

### 4.2. Conservation Implications

Despite the above listed limitations, important implications for effective conservation measures can be drawn from this study. As no clearly defined and uniform nesting season has been identified for the country, specific seasonal protective measures, such as temporal restrictions to beach access or increased monitoring, cannot be recommended.

The reported nesting activity indicates that the coastlines of Maldives could all be potential areas for nesting. As such, development projects should include careful planning and local ecological knowledge consultations prior to implementation. This applies to currently unknown nesting locations, but especially for documenting nesting hotspots such as L.Gaadhoo, which is under consideration for development [61].

Due to limited land availability in the country, coastlines and nesting beaches are in high demand for development and tourism activities. Balancing the ecological needs of sea turtles with human development is crucial for the long-term conservation of sea turtles.

The study has also identified new nesting hotspots and reinforced the importance of one previously known key nesting hotspot. However, further hotspots in areas currently underrepresented in the reporting of this study cannot be ruled out. We recommend the creation and implementation of a nationwide centralised data collection system, including training for dedicated personnel in each atoll to disseminate relevant information and collect data, including local ecological knowledge.

Green sea turtles are the dominant nesting species in the Maldives. Despite the recovery of this species on a global scale, the local decline of the Maldivian nesting population still warrants regional protection. For a comprehensive approach to protection, nesting as well as foraging habitats should be considered. Future studies could investigate the foraging areas utilised by green turtles nesting in the country as the habitat connectivity between them is currently unknown.

Hawksbill nesting in the Maldives is recorded in the north, and is rare, in contrast to higher reported historical numbers. As a critically endangered species, it is important to ensure that these nests are fully protected.

## 5. Conclusions

This study presents the first multi-year dataset on sea turtle nesting in the Maldives. During the study period 2018–2024, three species of sea turtles could be observed nesting in the Maldives, including greens, hawksbills and olive ridleys. Nesting numbers for these species vary greatly from historical reports, indicating declines for greens and hawksbills, while olive ridleys had not been recorded nesting in the Maldives before. The need for further monitoring efforts, especially those involving residential and uninhabited islands, has been identified. For the successful implementation of sustainability and management measures for sea turtles in the country, previously declared nesting hotspots should be further verified and new ones added if appropriate. While the nesting population in the Maldives might be small in comparison to other sea turtle nesting populations around the globe, it might provide interesting insights into climate resilience in the future.

## Figures and Tables

**Table 1:** Summary of recorded sea turtle nesting activity in the Maldives from 2018 to 2024 by atoll (in English alphabetical order) and species.

	Green			Hawksbill		Olive Ridley		Unknown		
Atoll	False Crawls	True Nests	NA	False Crawls	True Nests	False Crawls	True Nests	False Crawls	True Nests	NA
Alifu Alifu	1	3	0	0	0	0	0	0	0	0
Alifu Dhaalu	0	9	0	0	0	0	0	0	0	0
Baa	96	117	0	6	13	0	1	1	2	0
Dhaalu	34	23	1	0	0	0	0	0	0	0
Faafu	0	0	0	0	0	0	0	0	1	0
Fuvahmulah	0	1	0	0	0	0	0	0	0	0
Gaafu Alifu	0	0	0	0	0	0	2	0	0	0
Gaafu Dhaalu	0	0	0	0	0	0	0	0	3	0
Haa Alifu	1	0	0	0	0	0	0	0	0	0
Haa Dhaalu	0	1	0	0	2	0	5	0	0	0
Laamu	293	409	0	0	0	0	0	2	25	12
Lhaviyani	94	110	0	1	5	0	0	1	3	0
Noonu	438	316	0	3	7	0	0	24	26	0
North Male	6	8	0	1	3	0	0	0	0	0
Raa	39	69	4	7	14	0	4	3	3	1
Shaviyani	27	17	0	0	2	0	0	0	0	0
South Male	0	2	1	0	1	0	1	0	2	0
Thaa	0	1	0	0	0	0	0	0	0	0
Vaavu	1	0	0	0	0	0	1	0	0	0
TOTAL	1030	1086	6	18	47	0	14	31	65	13
Total by Species	2122			65		14		109		
%	91.86			2.81		0.61		4.72		

**Table 2:** Summary of green turtle hatching success % of all nests per atoll (in alphabetical order), including nest count (N), Minimum (min), Maximum (max), median and standard variation (SD).

Atoll	N	Min	Max	Median	SD
Alifu Alifu	2	0.00	55.20	27.60	39.03
Alifu Dhaalu	5	94.32	98.98	95.79	1.92
Baa	63	0.00	100.00	93.78	18.65
Dhaalu	3	94.00	96.15	95.00	1.08
Faafu	NA	NA	NA	NA	NA
Fuvahmulah	NA	NA	NA	NA	NA
Gaafu Alifu	NA	NA	NA	NA	NA
Gaafu Dhaalu	NA	NA	NA	NA	NA
Haa Alifu	NA	NA	NA	NA	NA
Haa Dhaalu	NA	NA	NA	NA	NA
Laamu	141	0.00	100.00	78.95	28.92
Lhaviyani	62	1.96	100.00	92.42	23.29
Noonu	136	17.27	100.00	92.01	18.18
North Male	6	43.40	99.07	94.79	21.40
Raa	26	0.00	99.04	87.24	40.85
Shaviyani	16	35.71	99.05	92.92	16.18
South Male	NA	NA	NA	NA	NA
Thaa	NA	NA	NA	NA	NA
Vaavu	NA	NA	NA	NA	NA

## Data Availability

The original contributions presented in this study are included in the Supplementary Material. Further inquiries can be directed to the corresponding author.

## Supplementary Materials

The following supporting information can be downloaded at https://www.mdpi.com/article/10.3390/ani16020307/s1, Supplementary Table S1: Complete 2018–2024 nesting dataset analysed in this study.
